# Current Synthesis and Systematic Review of Main Effects of Calf Blood Deproteinized Medicine (Actovegin^®^) in Ischemic Stroke

**DOI:** 10.3390/ijms21093181

**Published:** 2020-04-30

**Authors:** Florentina Carmen Firan, Aurelia Romila, Gelu Onose

**Affiliations:** 1 The Physical and Rehabilitation Medicine & Balneology Clinic Division—The NeuroRehabilitation Compartment, Teaching Emergency Hospital of the Ilfov County, 22104 Bucharest, Romania; firancarmen@yahoo.com (F.C.F.); 2 Medical Department, Faculty of Medicine and Pharmacy, “Dunarea de Jos” University of Galati, 800008 Galati, Romania; aurelia.romila@yahoo.com (A.R.); 3 The Gerontology and Geriatrics Clinic Department, Teaching County Emergency Hospital “Sf. Apostol Andrei”, 800578 Galati, Romania; 4 Physical and Rehabilitation MedicineDepartment, Faculty of Medicine, University of Medicine and Pharmacy “Carol Davila”, 020022 Bucharest, Romania; 5 The Neuromuscular Rehabilitation Clinic Division, Teaching Emergency Hospital ”Bagdasar-Arseni”, 041915 Bucharest, Romania

**Keywords:** ischemic stroke, pathophysiological/damage mechanisms, endogenous defense activity, pleiotropic action, deproteinized ultrafiltrate/hemodialysate compound, Actovegin^®^

## Abstract

Background: Stroke is one of the largest problems and clinical-social challenges within neurology and, in general, pathology. Here, we briefly reviewed the main pathophysiological mechanisms of ischemic stroke, which represent targets for medical interventions, including for a calf blood deproteinized hemodialysate/ultrafiltrate. Methods: We conducted a systematic review of current related literature concerning the effects of Actovegin^®^, of mainly the pleiotropic type, applied to the injury pathways of ischemic stroke. Results: The bibliographic resources regarding the use of Actovegin^®^ in ischemic stroke are scarce. The main Actovegin^®^ actions refer to the ischemic stroke lesion items’ ensemble, targeting tissue oxidation, energy metabolism, and glucose availability through their augmentation, combating ischemic processes and oxidative stress, and decreasing inflammation (including with modulatory connotations, by the nuclear factor-κB pathway) and apoptosis-like processes, counteracting them by mitigating the caspase-3 activation induced by amyloid β-peptides. Conclusion: Since no available therapeutic agents are capable of curing the central nervous system’s lesions, any contribution, such as that of Actovegin^®^ (with consideration of a positive balance between benefits and risks), is worthy of further study and periodic reappraisal, including investigation into further connected aspects.

## 1. Background

The term stroke is defined as ”…a neurological deficit attributed to an acute focal injury of the central nervous system (CNS) by a vascular cause, including cerebral infarction, intracerebral hemorrhage (ICH), and subarachnoid hemorrhage (SAH)…” [1], thus comprising an intraluminal obstructive/ischemic and/or wall tear and a lesion mechanism [2]. Generally, although with nuances mostly depending on the period when a specific study was undertaken, the geographic area examined, and the research methodology applied, a completed stroke is the most prevalent among major neurological conditions [3] and is the second-leading cause of death worldwide [4]. It has a marked potential to generate residual disability [5]; more precisely, “…almost two-thirds of stroke survivors leave hospital with a disability” [6]. These disabilities are quite often severe and/or permanent and account for the largest proportion of total disability-adjusted life years (DALYs), as the “largest contributor to this burden globally” [7], with a consequent socioeconomic impact [8], thus highlighting the severity of the overall impact of strokes. Strokes mainly affect the elderly, but “…approximately 10% of strokes occur in patients below 50 years of age” [9]. The increasing frequency of this condition in younger people represents a divergent trend from its global incidence and mortality, which seem to be diminishing [5,8,9].

“More women than men suffer strokes due to the risks of pregnancy, childbirth, and oral contraceptive use before age 30” [9]. Specifically, due to these risks, ischemic stroke prevails in younger patients and in elderly women, whereas it is more frequent in adult men, until reaching advanced (over 65 years—o.n.) age [10].

The total number of stroke events in the European Union (EU) was 613,148 in 2015 and is estimated to increase to 819,771 in 2035 [11], although the incidence and consequent mortality of strokes have had a descending trend since the early 2000s. Considerable differences exist between Eastern and Western Europe concerning the burden of stroke, which is significantly higher in Eastern European countries, including in terms of the specific mortality, which depends on better and faster treatment. This connects to the overall performance of each health care system, which is in turn linked with its level of financing, as well as with the effectiveness of public education campaigns to encourage an emergency response to stroke [11]. Romania ranks first among EU countries in both stroke incidence and mortality [11].

Regarding the main pathophysiological mechanisms of ischemic stroke targeted in this study, as preliminary considerations, preformed tissues for specific excitability, such as neurons, and most glial and striated muscle cells do not reproduce or replicate after a person is born [12,13]. Under injury conditions (e.g., ischemia in stroke), the central nervous system (CNS) reacts through preformatted pathways, which, for yet unknown reasons, exert active opposition to axonal regrowth and brakes to self-recovery within detrimental evolutive pathways [14].

A CNS insult entails, conditioned by complex, particular and not yet sufficiently deciphered mechanisms, a succession of local and regional damages. However, these damages have wide impacts from the intimate and genic on the body’s ensemble and systemic levels, which are classified as primary and secondary (events cascade) lesions [14,15,16,17,18]. Eventually, in total, the primary and secondary lesions contribute to neurological impairment [17]. 

Some lesion secondary developments overlap and are common for CNS conditions of different causes and partially comprise related biological pathways [19]. “Therefore, the concept of secondary CNS (including brain) injuries has become, especially in the last decades, the basis for developing an array of neuroprotective modern therapies in traumatic, ischemic, and degenerative injuries of the CNS (including both the brain and the spinal cord)” [15]. Thus, the drastic reduction of the cerebral blood flow by a sudden obstruction of predominantly large extracranial (vertebral/basilar, mostly internal, carotid) and/or intracranial (supplying or emerging from the Circle of Willis) arteries or small vessel(s) exposes the brain, the most dependent organ on oxygen metabolic consumption, to ischemia and deprivation of glucose provision. The latter appears to lower the brain tissue resistance to hypoxia; if such a severe interruption lasts more than five minutes without enough flux compensation through collateral circulation variants, it results in irreversible damage and consequent large brain infarction [20,21,22,23]. The occlusion is caused in most cases by thrombosis/thrombus formation in the atherosclerotic plaques (prominent in the vascular lumen(s)), which characterizes atherosclerotic cerebrovascular disease, microatheroma, lipohyalinosis (related to small, deep vessel thromboses, resulting more often in cerebral lacunar infarct lesions), embolism, hemodynamic severe disturbance leading to cerebral hypoperfusion, or, in rarer cases, local inflammatory conditions, like vasculitis [20,23,24]. The atherosclerotic lesions of the vascular walls are also considered to be of inflammatory origin: leukocyte local infiltration, proinflammatory cytokines, and adhesion molecule release, which favor monocyte and T-lymphocyte endothelial adherence and lead to subsequent penetration and maintenance of a continuous inflammatory status [23,25] and/or a systemically infectious origin (*Chlamydia pneumoniae*, Cytomegalovirus, and *Helicobacter pylori*) [25]. Consequently, after blood supply arrest, a succession of extremely complex and intertwined pathophysiological processes begins within seconds [10], both detrimental and as part of the recovery, which may continue for weeks, months, or years, until reaching a clinical-evolutive relative plateau [26]. These processes are emphasized briefly below.

Abrupt and relatively prolonged deprivation of blood flow, i.e., oxygen and energetic (basically, glucose) support, leads to production collapse and drastic shortage, especially of the metabolically produced principal molecular storage and energy provider, ATP, in the most-affected brain tissue. Such severe biochemical injury generates an important amount of direct necrotic cell deaths in the core of the ischemic zone, in part because the membranes’ functional and structural integrity can no longer be sustained. Being energy-dependent, resting and excitation neuronal states, which are both membrane-active processes, are markedly altered, resulting in local still-living cells dying or being at increased risk of dying (although this may be remedied if irrigation is restored sufficiently quickly). This collateral perfusion occurs in the ischemic penumbra of the infarct’s periphery: (1) in neural control disturbance/abolishment of various types and severities and over different directly and/or indirectly connected territories and (2) in enhanced, inappropriate, and detrimental inner bio-pathologic augmented activity with enhanced ATP consumption, which is already diminished [10,19,27]. 

If the blood flow arrest continues without sufficient collateral flux supply and within cerebrovascular autoregulation [22] or in the peripherally situated ischemic penumbra, further injuries may occur, including, at the intimate level, disturbance of mitochondrial functionality with a consequently imbalanced ratio between pro- and antioxidant factors (including related scavengers) in favor of the former). Oxidative (or nitrosative) stress is mainly generated by the highly enhanced production of reactive oxygen species (ROS), generally associated with depletion but with time-dependent sequential nuances, instead providing a gene-coded transcription-factor-mediated activation of an endogenous-related defense capability. The antioxidant-response elements (AREs) of antioxidants, such as l-c-γ-glutamyl-l-cysteinylglycine (glutathione (GSH)), are highly important [28,29,30,31,32,33,34]. Subsequently, lipid peroxidation, together with phospholipases, also affect the membranes’ integrity. Other critical damage actions of ROS include augmentation of the Ca^2+^ intracellular amount, cytoskeleton, and DNA insults with protein oxidation [35,36], proclivity to secondary misfolding [19], enhanced involvement by gene expression activation of nuclear factor-κβ (NF-κB) of proinflammatory cytokines (chemokines and interleukins) and adhesion molecules (expressed by activated endothelial cells, which attract and stimulate the tissue plasminogen activator (t-PA) and are also considered to have therapeutic capabilities, as recombinant tissue plasminogen activator; rtPA) [37,38], and the stimulation of matrix metalloproteinases (MMPs) and other (metallo) proteases [22,39].

First, soon after ischemia is installed, neutrophils infiltrate (chemotactism) and injure the blood–brain barrier (BBB). A modern, expanded, more complex, and related conceptual structuring is the neurovascular unit that physiologically entails, by cell–cell signaling and interactions, the coordinated and efficacious comprehension and functioning of the BBB location, neurons, microglia, astrocytes, pericytes, endothelial smooth muscle cells, and intrinsic matrix proteins, and which has the adaptive capability to dynamically modify itself according to and within morphological-functional changes during post-stroke partial recovery [40,41]. Subsequently, macrophages and lymphocytes, including T cytotoxic (natural killer; NK) and B types [36], enter the damaged cerebral tissue within the above-mentioned inflammation context. In addition to those already noted, the related primum movens dwells in the signals represented by the modified osmolarity [42] and consistency of the slack post-occlusion blood, addressed to the local endothelial structure and thrombocytes [37]. Additionally involved in different but interlinked pathophysiological-related sequences are leukotrienes; growth factors; prostaglandins; astrocytes; further cell adhesion molecules, e.g., selectins; intercellular adhesion molecule 1 (ICAM-1); vascular cell adhesion molecule 1 (VCAM-1); and integrins [10,17,22,36], including with and through microglial cells that are resident in the CNS and partially transformed into phagocytes [37]. Different inflammatory pathways, some of which are respiratory [19], are also stimulated by accumulation of necrotic debris in the focal ischemic zone [39,43].

Consequent to hypoxia, the complex pathophysiological context of the ischemic stroke partially and briefly outlined above also entails acidosis, which is metabolically induced in local hypo- or anoxic circumstances, with the accumulation of lactate and hydrogen ions (H^+^); the latter stimulates the production of ferrous iron-mediated ROS [10,27,36,38]. The major pathways for cell deaths are apoptosis (type I) and apoptosis-like/anoikis, autophagy (type II), and necrosis (type III) [19,41,44]. 

”Brain infarction was traditionally considered to be a classic example of liquefactive necrosis” [45] that can supervene quickly and brutally within a few minutes after severe and prolonged brain ischemia in the cerebral tissue, which has low tolerance to hypoxemia, such that necrosis is prone to be augmented by further pathophysiological mechanisms [46] via osmolar overload and consequent osmolysis, especially if suddenly installed [19,42]. However, a similar irreversible outcome, i.e., cell death, may also result following the other linked pathophysiological secondary injury events (summarized above) but more slowly. These latter delayed deadly damages nonetheless offer a time window for the at-risk biological structures to be rescued, at least partially [10], by spontaneous processes (prompt reperfusion, mainly based on efficient collateral blood supply restoration and vessel repermeabilization) and/or interventions. Within a major ischemic stroke, except for the overall successfully achieved (rtPA) thrombolysis, such favorable inner natural evolutions or outcomes usually do not prevail. Thereby, apoptosis and apoptosis-like phenomena also occur, including concomitantly. The former, apoptosis, is the classic pattern of programmed cell death. It often entails the mediated destruction of caspases via its propensity for phagocytosis cells to break up in connection with nuclear condensation. This may run on the intrinsic [10,29,45] mitochondrial pathway based on the release signaling of cytochrome c (a key component in the respiratory chain) and endonuclease G by proteins such as Bad, Bak, Bax, Bid, and Bim and/or those involved in metabolic pathway regulation and membrane lipids. Apoptosis may also involve permeability transition pore openings in the inner membrane components that mainly contribute to the mitochondrial outer membranes permeabilization (MOMP) [36,45,47,48]. Apoptosis targets connected enzymes such as poly-ADP-ribose-polymerase (PARP), which is, with important sex differences in its effects and with consequent nuclear DNA damage and/or exit from the mitochondria, entrance into the intracellular fluid, and continued translocation into the nucleus of the apoptosis-inducing factor (AIF), considered as being caspase-independent [36,49].

The extrinsic pathway is initiated by suicidal molecular signals such as lethal ligands or death ligand trimer, responsible for the ligation to the cell external surface of death receptors, for tumor necrosis factor (TNF)-related apoptosis-inducing ligands (TRAIL), such as tumor necrosis factor α (TNF-α), the human diploid fibroblast (FS-7) cell-line-associated surface antigen, (Fas)/Apoptosis antigen, Apo-1 (Cluster of Differentiation (CD95)), and death receptor 4 (DR4) [10,36,49,50,51,52]. Eventually, all these pathophysiological mechanisms lead to cellular dysfunction. Apoptosis and apoptosis-like forms are relatively different concerning the related changes in the nuclear structure: apoptosis involves “…stage II chromatin condensation into compact figures…”, whereas apoptosis-like involves “…less-compact chromatin condensation” (stage I) [47].

An additional pathway, named anoikis, involves the detachment of cells from the extracellular matrix (ECM) [53], including mainly with “…MMP-induced proteolysis of the neurovascular matrix …” [41]. This may also lead to programed cell death soon after stroke onset, consequent to the BBB deterioration. Specifically, this occurs due to the neurovascular unit’s morphological impairment, and its secondary disfunction regards the signaling of the related inter-cells with their ECM [19,41].

Notably, in brain ischemia, necrosis, and different types of apoptosis/programed cell death, the same neuron may be affected simultaneously by caspases, calpains, and cathepsins [45].

Considering the vast complexity of CNS lesions, including in post-ischemic stroke, a justified goal for their treatment is drugs able to efficiently protect against the above-described disastrous injury developments and to provide compensatory and even recovery stimulation capabilities, as detailed below. 

## 2. Methods

Given the justification noted above, an available pharmacological neuro-/bio-trophic compound with pleiotropic action, applicable to the treatment of ischemic stroke, is the calf blood deproteinized ultrafiltrate/hemodialysate, Actovegin^®^ (Takeda Austria GmbH, Linz, Austria).

Our goal was to target and elucidate Actovegin^®^’s well-known beneficial and therapeutic effects that either directly or indirectly interfere with the morbidity pathways of ischemic stroke. These were expressly selected and synthesized in Section 1 within the complex and complicated intermingling between the damage mechanisms (DMs) [19,46] and endogenous defense activity (EDA) [54] (see Section 4).

**Figure 1:**
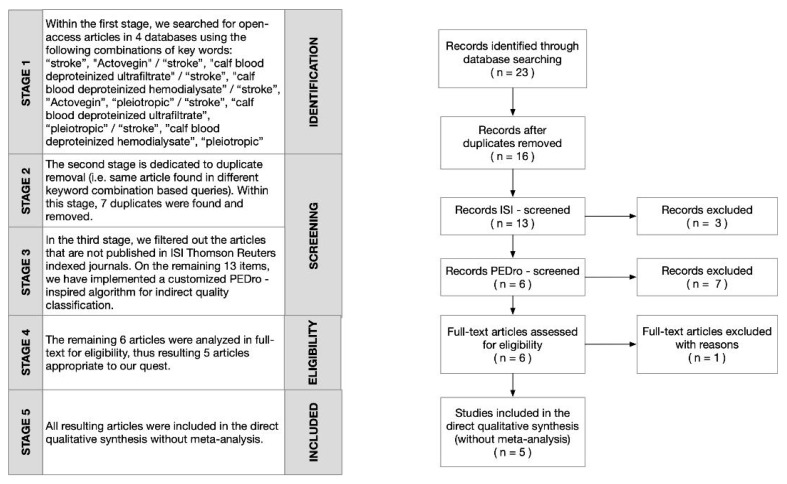
Adapted from [59], Preferred Reporting Items for Systematic Reviews and Meta-Analyses (PRISMA) flow diagram, customized for our study.

Accordingly, we conducted a systematic literature review regarding the use of Actovegin^®^ in stroke. For this purpose, we searched for and interrogated related articles for the period 1 January, 2001–31 December, 2019 in reputable international medical databases: National Center for Biotechnology Information (NCBI)/PubMed, NCBI/PubMed Central (PMC) [55], Elsevier [56], Physiotherapy Evidence Database (PEDro) [57], and Institute for Scientific Information (ISI) Web of Knowledge/Science [58] (via ISI Thomson Reuters index check). In searching, we used specific sets of keywords: (“stroke”, “Actovegin”/“stroke”, and “calf blood deproteinized hemodialysate”/“stroke”, “calf blood deproteinized ultrafiltrate”/“stroke”, “Actovegin”, “pleiotropic”/“stroke”, “calf blood deproteinized hemodialysate”, “pleiotropic”/“stroke”, “calf blood deproteinized ultrafiltrate”, and “pleiotropic”). Table 1 shows the numerical results of our search, which was based on a focused, step-by-step classification according to the stages of the largely used literature identification and selection method, Preferred Reporting Items for Systematic Reviews and Meta-Analyses (PRISMA) (see Figure 1) [59].

Within the PRISMA paradigm, we considered only open-access/free full-text articles written in English and indexed in the ISI Thomson Reuters database. To quantify the scientific impact/indirect quality classification of each of the articles remaining after ISI checking and duplicates were removed, a custom dedicated evaluation algorithm was used [60]: first, the year of publication was considered together with the total number of citations to compute the average number of citations per year; secondly, the final PEDro score [57] for each article considered was calculated via a weighted average formula; lastly, we retained only those articles that obtained a score of at least 4 (see Table 2 for details). Thus, we eventually found five articles whose content was closest to our query, revealing that works regarding Actovegin^®^ and its use in stroke treatment are rather scarce; therefore, the subject matter we have chosen is worthy of investigation.

Notably, we read all of the articles identified by keywords, even if they did not qualify according to our customized PEDro-inspired indirect quality classification (see Table A1). In addition, we purchased articles that were not open-access/free full-text but that contained valuable related information to include in the study.

Despite the systematic review rigorous selection filter we have applied, according to the above-mentioned criteria-based classification methodology, some of the works related to this subject still might be missed [60]. Thus, with the aim for the search to be as exhaustive as possible, we used a mixed course of collecting the necessary bibliographic resources via standardized (the systematic literature review) and nonstandardized methods (i.e., considering related papers, including in the Romanian language) (see Table A2).

## 3. Results

Actovegin^®^ is a deproteinized, antigen- and protein-free hemoderivative/hemodialysate obtained from calf blood through two ultrafiltration stages, resulting in a complete deproteinized complex of more than 200 bioactive components with molecular weight less than 5000 Da [62,66,67,68], thus small enough to cross the BBB [69]. Its analysis using the chromatography technique showed that it is a mixture encompassing (alphabetically): adenosine monophosphate (AMP); amino acids (25.8%); biogenic amines (2.2%) and polyamines; choline; eicosanoids; and electrolytes (e.g., calcium, chloride, magnesium, phosphate, sodium, and potassium); components of the cellular membranes (glycosphingolipids); inositol-phospho-oligosaccharides (IPOs, the main bioactive molecules); hexose (38.5%); lactate (21.2%); succinate; and vitamins (11%) [66,67,69,70,71]. This large bundle of substances enables covering interferences with a wide spectrum of biomolecular (including pathological) pathways, forming the basis of its pleiotropy and perhaps its multipotential [17] action, but means that it is complicated and simultaneously divergent from its conceptual paradigm of use (i.e., as a composed yet unitary medicine). Identifying the effects of each compound separately within it is thus difficult [67]. 

Studies undertaken since the 1960s [71] on Actovegin^®^ emphasized its biotrophic actions, including on skin and subcutaneous tissue lesions and/or those consequent to favorably influencing sanguine flow [66]. More recent literature, mainly since the early 2000s, also highlighted the beneficial effects of this drug on the different pathological pathways of neurological and/or vascular diseases. It “was recently demonstrated to have neuroprotective effects on neurons by increasing neuron and synaptic numbers…” [66]. In vitro studies indicated possible neuroregenerative effects by increasing the neurite length and the number of neuronal and excitatory synaptic contacts [62,67,69,72].

Based on studies of animal models, the actions of Actovegin^®^ were documented at the intimate level, including its interference with some important pathways of ischemic stroke pathophysiology and other conditions of the nervous system, either traumatic or degenerative, and central and peripheral, including diabetic polyneuropathy and endothelial dysfunction [62,65,66,72].

In brief, this calf blood deproteinized ultrafiltrate augments tissue oxidation, energy metabolism, and glucose availability, thus combating ischemic processes [66]. Therefore, it may protect against hypoxic cell injury [62,72] and acidosis by lactate accumulation [63]. It also counteracts oxidative stress, inflammation (by subtle interference with the NF-κB pathway), and apoptosis processes through mitigating the caspase-3 activation induced by amyloid β-peptides (Aβs) [62,65,66,67,69].

In more detail, Actovegin^®^ improves tissue oxygen and glucose consumption and energy production, for instance, in the hippocampus area, which is linked mainly to functions such as spatial learning and memory [73]. The main consequence is the increase in the related cellular metabolism by enhancing mitochondrial capacity, which has a positive effect on glucose carrier activity and oxidation, related to pyruvate dehydrogenase [62,66,67,70,72]. Thus, Actovegin^®^ also has insulin-like activity (ILA) [72]; IPOs may positively regulate glucose use through the activity of BBB transporters (i.e., the uniporter proteins glucose transporter 1 (GLUT1) and glucose transporter 4 (GLUT4) [66,70]. 

Oxidative stress is decreased by Actovegin^®^ [61,62,65,66,69,73] through inhibiting the nuclear enzyme poly ADP ribose polymerase (PARP) [66,68], which can detect and repair DNA damage under normal conditions but can simultaneously compromise glycolysis and mitochondria respiration, leading eventually to cellular death [66]. Using in vitro studies of rat primary hippocampal neurons, Elmlinger et al. [62] reported beneficial effects of this drug via diminishing their inner level of ROS. This dose-dependent effect was also found in in vivo studies after treatment with Actovegin^®^ [62,66,67,73]. 

Actovegin^®^ is involved in inflammation pathways though the activation/modulation of NF-κB with, in addition to the above-mentioned detrimental actions, a beneficial role in neuroprotection mediated by proinflammatory cytokines such as TNF-α [65,66,69,73].

Calf blood deproteinized ultrafiltrate reduces the apoptosis processes induced by beta amyloid peptides (Aβ_25–35_) [62,66,67]. This effect, first studied in vitro [62], was observed through a dose-dependent decrease in activated caspase-3 in treatment with Actovegin^®^ [62,63,66,67,69]. More specifically, Actovegin^®^’s antiapoptotic and neuroprotective effects were demonstrated in a rat model with global cerebral ischemia, induced by reducing cell death in hippocampal Cornu Ammonis (CA1) areas [66,73].

In the last decade, this calf blood deproteinized hemodialysate’s beneficial effects were studied in trials targeting chronic conditions such as diabetic polyneuropathy [72] and post-stroke cognitive impairment (PSCI) [63]. Notably, “stroke survivors are at increased risk of developing cognitive impairment” [61].

The concept of disease modifiers in vascular cognitive impairment (VCI) was highlighted within the 9th International Congress on Vascular Dementia in Ljubljana, Slovenia, 2015 [64]. According to one of its conclusions, Actovegin^®^ can be included in the above-mentioned category of pharmacological agents, including as an adequate medicine for the treatment of this mental disturbance [61,64,67].

Type 2 diabetes, diabetic polyneuropathy, and stroke should be mentioned due to their clinical importance and their frequent coexistence and related complex therapeutic approaches. Diabetes mellites (DM) is associated with an increased risk of mild cognitive impairment, dementia, and stroke [65]. Briefly, although Actovegin^®^’s beneficial action pathways in diabetic polyneuropathy are not yet sufficiently understood, in addition to the factors noted above, the literature refers to its capability to “improve the cellular energy level, enhance glucose uptake and metabolism” [72]; foster ”stimulation of glucose transport, pyruvate dehydrogenase, and glucose oxidation…” [62]; and “increase oxygen absorption and utilisation” [72].

Considering that epilepsy is a possible, mainly delayed, complication after stroke [74], Actovegin^®^ is a neuro-/bio-trophic for which such a condition is not among the contraindications [75].

## 4. Discussion

A modern paradigm for medication related to the above analysis asserts that “…using neuroprotective molecule with only one mechanism of action in disease treatment is a utopist idea…” [46]. Therefore, it is currently considered to be possible to provide better outcomes using therapeutic agents with pleiotropic or multimodal actions, although it is difficult to establish an immutable border in multipotential medicines between such properties [17], especially given the dynamic understanding of their effects due to a growing knowledge about this complicated domain. Specifically, “…a neuroprotective pleiotropic effect is related to DM (o.n.: i.e., the bundle of lesion events, as summarized above, ranging from excitotoxicity—and more—to individual genetic particularities of the post-insult response [19,46]) and represents the capacity of a pharmacological agent to interfere in more than one pathophysiological process” [54], whereas a multimodal effect refers to a drug’s “…capacity to simultaneously regulate, in the post-lesional brain, two or more endogenous neurobiological processes of EDA…” [54] (i.e., mainly encompassing four basic neurobiological continuous functions within the nervous system—neuroprotection, neurotrophicity, neuroplasticity, and neurogenesis—in both physiological (especially for the latter three) and pathological circumstances). 

The subtle and insufficiently deciphered interferences and partial overlaps between EDA and the not exclusively detrimental (see below) consequences of DM should be specified. This includes some EDA components; for instance, excess neuroplasticity may result in neuropathic pain, movement disorders, etc. [46]. Another example is inflammation that seems to act ambivalently and participate in “…oligodendrocyte cell death and contribute to demyelination after stroke or TBI…” [76]. However, it also has a regenerative connotation, stimulating remyelination, and neurogenesis, angiogenesis, synaptogenesis, and axonal sprouting [77]. 

Concerning the sophisticated conditionings between EDA components, we briefly note some more recently emphasized connections and differences (perhaps competing) within the time frame of the progress of post-insult brain tissue reactions. Neuroprotection prevails in the first hours or days, opposing “cell death, inflammation, and scarring” ([26] and [78]; the latter is followed by “tissue reorganization” [78]. It is also partially overlapped at its inception after an elapsed period of tens of hours to a few days, with neuroplasticity and the functions of neurotrophicity and neurogenesis, as much as possible in this difficult and biologically challenging and harsh context. These are all intermingled between and with DM evolutions [46,54] toward “improving impairment/disability” [78] and “function” [26]. This extends over weeks, months, and, potentially, years [26,78], essentially referring to rehabilitation and neural repair in the subacute phase and to goal-specific training and repair in the chronic phase [78].

## 5. Conclusions

At present, no treatment intervention is available to definitively heal CNS lesions [79,80]. However, affected patients need the best possible clinical management, even under the range of weak current therapeutic options. Such patients include those affected by the frequent and disabling pathology domain represented by stroke. Therefore, further study of the literature is warranted of the possible use of any therapeutic agents that have produced favorable, albeit limited, results, including those providing small functional gains [81]. Amongst the treatments deserving further study and periodic reassessment is the Actovegin^®^ compound, which is a pharmacological medicine with ”…pleiotropic, neuroprotective, and metabolic effects…” that “…fits this future vision of an integrated treatment paradigm” [66], related possible multimodal therapeutic effects being also worth being checked going forward.

## Figures and Tables

**Table 1:** Keywords sets-based search for related articles—numerical results. PMC: PubMed Central and PEDro: Physiotherapy Evidence Database.

Keywords/Database	Elsevier	PubMed	PMC	PEDro
“stroke”, “Actovegin”	0	0	16	0
“stroke”, “calf blood deproteinized ultrafiltrate”	0	0	0	0
“stroke”, “calf blood deproteinized”, “hemodialysate”	0	0	0	0
“stroke”, “Actovegin”, “pleiotropic”	0	0	7	0
“stroke”, “calf blood deproteinized ultrafiltrate”, “pleiotropic”	0	0	0	0
“stroke”, “calf blood deproteinized”, “hemodialysate”, “pleiotropic”	0	0	0	0
**Total**	**0**	**0**	**23**	**0**

**Table 2:** Indirect quality classification for the selected articles. ISI: Institute for Scientific Information.

No.	Title	Authors	ISI	Year	Citations	Citations/Year	PEDro
1	Post-Stroke Dementia—A Comprehensive Review	Mijajlović et al. [61] (in the body text—see further)	Yes	2017	78	26	10
2	Neuroprotective and Anti-Oxidative Effects of the Hemodialysate Actovegin^®^ on Primary Rat Neurons in Vitro	Elmlinger et al. [62] (in the body text—see further)	Yes	2011	124	~14	8
3	ARTEMIDA Trial (A Randomized Trial of Efficacy, 12 Months International Double-Blind Actovegin^®^): A Randomized Controlled Trial to Assess the Efficacy of Actovegin^®^ in Poststroke Cognitive Impairment	Guekht et al. [63] (in the body text—see further)	Yes	2017	33	11	6
4	Towards the Concept of Disease-Modifier in Post-Stroke or Vascular Cognitive Impairment: A Consensus Report	Bordet et al. [64] (in the body text—see further)	Yes	2017	23	~8	4
5	Diabetes and the Brain: Issues and Unmet Needs	Bornstein et al. [65] (in the body text—see further)	Yes	2014	43	7	4

## Appendix A

**Table A1:** ISI-Reuters database indexed articles.

Article	Publ. Year	No. of Citations	No. of Citations/Year	PEDro Score
Mijajlović et al., Post-stroke dementia—A comprehensive review, *BMC Med. 2017; 15: 11. Published online 2017 Jan 18. doi:10.1186/s12916-017-0779-7*	2017	78	26	10
Elmlinger et al., Neuroprotective and Anti-Oxidative Effects of the Hemodialysate Actovegin^®^ on Primary Rat Neurons in Vitro, *Neuromolecular Med. 2011 Dec; 13(4): 266–274.* *Published online 2011 Oct 9. doi:10.1007/s12017-011-8157-7*	2011	124	13.78	8
Guekht et. al, ARTEMIDA Trial (A Randomized Trial of Efficacy, 12 Months International Double-Blind Actovegin^®^): A Randomized Controlled Trial to Assess the Efficacy of Actovegin in Poststroke Cognitive Impairment, *Stroke. 2017 May; 48(5): 1262-1270. Published online 2017 Apr 24.doi:10.1161/STROKEAHA.116.014321*	2017	33	11	6
Bordet et al., Towards the Concept of disease-modifier in post-stroke or vascular cognitive impairment: a consensus report, *BMC Med. 2017; 15: 107. Published online 2017 May 24. doi:10.1186/s12916-017-0869-6*	2017	23	7.66	4
Liu et al., Curcumin Protects against Ischemic Stroke by Titrating Microglia/Macrophage Polarization, *Front Aging Neurosci. 2017; 9: 233. Published online 2017 Jul 21. doi:10.3389/fnagi.2017.00233*	2017	22	7.33	4
Borstein et al., Diabetes and the brain: issues and unmet needs, *Neurol Sci. 2014; 35(7): 995–1001. Published online 2014 Apr 29. doi:10.1007/s10072-014-1797-2*	2014	43	7.17	4
Meilin et al., Treatment with Actovegin improves spatial learning and memory in rats following transient forebrain ischaemia, *J Cell Mol Med. 2014 Aug; 18(8): 1623–1630. Published online 2014 May 6. doi:10.1111/jcmm.12297*	2014	31	5.17	3
Voskoboeva et al., Homocystinuria due to cystathionine beta-synthase (CBS) deficiency in Russia: Molecular and clinical characterization, *Mol Genet Metab Rep. 2018 Mar; 14: 47–54. Published online 2017 Dec 27. doi:10.1016/j.ymgmr.2017.11.001*	2018	7	3.5	2
Dong et al., Cerebrolysin improves sciatic nerve dysfunction in a mouse model of diabetic peripheral neuropathy, *Neural Regen Res. 2016 Jan; 11(1): 156–162. doi:10.4103/1673-5374.175063*	2016	12	3	2
Krasnov et al., Early Aging in Chernobyl Clean-Up Workers: Long-Term Study, *Biomed Res Int. 2015; 2015: 948473. Published online 2015 Jan 27. doi:10.1155/2015/948473*	2015	10	2	1
Mattyasovszky et al., Exposure to radial extracorporeal shock waves modulates viability and gene expression of human skeletal muscle cells: a controlled in vitro study, *J Orthop Surg Res. 2018; 13: 75. Published online 2018 Apr 6. doi:10.1186/s13018-018-0779-0*	2018	4	2	1
Zhang et al., Isosteviol Sodium Protects against Ischemic Stroke by Modulating Microglia/Macrophage Polarization via Disruption of GAS5/miR-146a-5p sponge, *Sci Rep. 2019; 9: 12221. Published online 2019 Aug 21. doi:10.1038/s41598-019-48759-0*	2019	0	0	0
Zhang et al., Progression in Vascular Cognitive Impairment: Pathogenesis, Neuroimaging Evaluation, and Treatment, *Cell Transplant. 2019 Jan; 28(1): 18–25. Published online 2018 Nov 29. doi:10.1177/0963689718815820*	2019	0	0	0

**Table A2:** Articles not indexed ISI-Reuters database.

Article
Guekht et al., A Randomised, Double-Blind, Placebo-Controlled Trial of Actovegin^®^ in Patients with Post-Stroke Cognitive Impairment: ARTEMIDA Study Design, *Dement Geriatr Cogn Dis Extra. 2013 Jan-Dec; 3(1): 459-467. Published online 2013 Dec 14. doi:10.1159/000357122*
Khandelwal et al., Cardiovascular autonomic functions & cerebral autoregulation in patients with orthostatic hypotension, *Indian J Med Res. 2011 Oct; 134(4): 463**–469*
Onose et al., Integrative emphases on intimate, intrinsic propensity/ pathological processes-causes of self-recovery limits and also, subtle related targets for neuroprotection/ pleiotropicity/ multimodal actions, by accessible therapeutic approaches-in spinal cord inj, *J Med Life. 2010 Aug 15; 3(3): 262**–274. Published online 2010 Aug 25*
